# CTHRC1 promotes angiogenesis by recruiting Tie2-expressing monocytes to pancreatic tumors

**DOI:** 10.1038/emm.2016.87

**Published:** 2016-09-30

**Authors:** Jaemin Lee, Jinhoi Song, Eun-Soo Kwon, Seongyea Jo, Min Kyung Kang, Yeon Jeong Kim, Yeonsil Hwang, Hosung Bae, Tae Heung Kang, Suhwan Chang, Hee Jun Cho, Song Cheol Kim, Seokho Kim, Sang Seok Koh

**Affiliations:** 1 Aging Research Institute, Korea Research Institute of Bioscience and Biotechnology, Daejeon, South Korea; 2 Department of Biological Sciences, Dong-A University, Busan, South Korea; 3 Department of Biological Sciences, Korea Advanced Institute of Science and Technology, Daejeon, South Korea; 4 Graduate School of Medical Science and Engineering, Korea Advanced Institute of Science and Technology, Daejeon, South Korea; 5 Department of Immunology, School of Medicine, Konkuk University, Seoul, South Korea; 6 Department of Biomedical Sciences and Physiology, Asan Medical Center, University of Ulsan School of Medicine, Ulsan, South Korea; 7 Immunotherapy Convergence Research Center, Korea Research Institute of Bioscience and Biotechnology, Daejeon, South Korea; 8 Department of Surgery, Asan Medical Center, University of Ulsan School of Medicine, Ulsan, South Korea

## Abstract

CTHRC1 (collagen triple-helix repeat-containing 1), a protein secreted during the tissue-repair process, is highly expressed in several malignant tumors, including pancreatic cancer. We recently showed that CTHRC1 has an important role in the progression and metastasis of pancreatic cancer. Although CTHRC1 secretion affects tumor cells, how it promotes tumorigenesis in the context of the microenvironment is largely unknown. Here we identified a novel role of CTHRC1 as a potent endothelial activator that promotes angiogenesis by recruiting bone marrow-derived cells to the tumor microenvironment during tumorigenesis. Recombinant CTHRC1 (rCTHRC1) enhanced endothelial cell (EC) proliferation, migration and capillary-like tube formation, which was consistent with the observed increases in neovascularization *in vivo*. Moreover, rCTHRC1 upregulated angiopoietin-2 (Ang-2), a Tie2 receptor ligand, through ERK-dependent activation of AP-1 in ECs, resulting in recruitment of Tie2-expressing monocytes (TEMs) to CTHRC1-overexpressing tumor tissues. Treatment with a CTHRC1-neutralizing antibody-abrogated Ang-2 expression in the ECs *in vitro*. Moreover, administration of a CTHRC1-neutralizing antibody to a xenograft mouse model reduced the tumor burden and infiltration of TEMs in the tumor tissues, indicating that blocking the CTHRC1/Ang-2/TEM axis during angiogenesis inhibits tumorigenesis. Collectively, our findings support the hypothesis that CTHRC1 induction of the Ang-2/Tie2 axis mediates the recruitment of TEMs, which are important for tumorigenesis and can be targeted to achieve effective antitumor responses in pancreatic cancers.

## Introduction

Pancreatic ductal adenocarcinoma is one of the most aggressive and lethal malignancies in the world, with a mean survival of ~5 months and a 5-year survival rate of ~14%.^1^ More than 80% of patients present with late-diagnosis, metastatic disease and often exhibit resistance to conventional chemotherapy and radiotherapy. Despite numerous ongoing clinical trials, pancreatic cancer has been shown to be highly refractory to combined treatment with gemcitabine and other therapeutics as well as newly developed agents. Thus, there is a critical need for novel therapeutic strategies or targets to reduce mortality and increase the overall survival rate of patients with pancreatic cancer.

CTHRC1 (collagen triple helix repeat containing-1), a secreted protein that has hormone-like characteristics, is known to be involved in tissue remodeling processes affecting vascularity and bone formation. CTHCR1 is expressed in injured arteries and participates in remodeling the adventitia of injured vessels by promoting cell migration and inhibiting collagen synthesis in fibroblasts and smooth muscle cells.^2^ Recent studies have reported a close correlation between CTHRC1 expression and the aggressiveness of various human cancers. A human tumor complementary DNA array analysis showed that the *CTHRC1* gene is expressed in the majority of human solid cancers, and a transcriptome analysis identified *CTHRC1* among genes that are differentially expressed in breast lobular carcinoma versus normal ductal and lobular cells.^3, 4^ Upregulation of CTHRC1 was associated with invasive and metastatic melanomas but not with benign nevi or non-invasive specimens; moreover, *in vitro* migration of melanoma cancer cells was decreased by inhibiting CTHRC1 expression.^3^ Most dermatofibrosarcoma protuberans, locally aggressive neoplasms that frequently metastasize, are also positive for CTHRC1 expression, whereas most dermatosarcomas, a common benign fibrohistiocytic tumor, are not.^5^ CTHRC1 expression is significantly higher in breast cancer than in normal tissues or precursor lesions and is correlated with the risk of bone metastasis.^6^ Recently, we reported that upregulation of CTHRC1 is related to the progression and metastasis of pancreatic cancers through the activation of several key signaling molecules, including Src, focal adhesion kinase, paxillin, mitogen-activated protein kinase (MEK), extracellular signal-regulated kinase (ERK), and Rac1.^7^ Thus far, the role of CTHRC1 as an autonomous activator in tumor cells is well known, but little information on the biological properties of CTHRC1 in the tumor microenvironment is available.

The tumor microenvironment is composed of a mixture of extracellular molecules and several types of cells, including tumor cells, endothelial cells (ECs), fibroblasts, and immune cells. The consequent proinflammatory tumor microenvironment affects vascular activity in the form of angiogenesis, which supports tumor growth and metastasis. Angiogenesis is a hallmark of tumorigenesis in the tumor microenvironment and allows the tumor to expand beyond the limits of oxygen and nutrient perfusion and eventually metastasize to distant organs.^8^ During physiological angiogenesis, new blood vessels are formed through a well-orchestrated series of events that include the recruitment of perivascular support cells and the formation of a functional lumen.^9^ A recent study noted the close interaction that occurs between cells of the innate immune system and the developing vascular network during tumor angiogenesis.^10^ The critical interactions between immune cells and tumor angiogenesis have led to the suggestion that targeting tumor-infiltrating immune cells may represent a viable anti-angiogenic strategy for cancer treatment.^11^ Recently, a subset of monocytes expressing Tie2, an angiopoietin receptor, have been shown to have a particularly important role in tumor angiogenesis. Tie2 expression was previously thought to be predominantly restricted to ECs and hematopoietic stem cells. However, Tie2-expressing monocytes (TEMs), a subpopulation of circulating, tumor-infiltrating myeloid cells with a highly proangiogenic phenotype, have been found in both humans and mice.^12^ Angiopoietin 2 (Ang-2), a Tie2 ligand, is overexpressed by ECs in tumors, further augmenting the ability of TEMs to stimulate angiogenesis through upregulation of proangiogenic enzymes, such as thymidine phosphorylase and cathepsin B.^13, 14^

Previous reports have suggested that CTHRC1 secreted by tumor cells acts in an autocrine manner to modulate tumor progression and metastasis. However, the angiogenetic function of CTHRC1 in the tumor microenvironment remains unclear. Here we found that CTHRC1 is closely associated with tumor vascularization in pancreatic cancers. Treatment with recombinant CTHRC1 (rCTHRC1) promoted EC activation and secretion of Ang-2 through ERK-dependent nuclear translocation of AP-1 (activator protein-1). Moreover, elevated levels of Ang-2 facilitated infiltration of TEMs into CTHRC1-overexpressing tumor tissues. These results were further supported by the correlation between CTHRC1-induced Ang-2 expression in ECs and TEM infiltration into the tumor tissues, which was demonstrated by injection of a CTHRC1-neutralizing antibody into Pancreatic ductal adenocarcinoma models. These findings suggested that CTHRC1 blockade may inhibit primary tumorigenesis and metastasis by reducing vascular progression in pancreatic cancers.

## Materials and methods

### Cell lines

The human pancreatic cancer cell lines MiaPaCa-2, CFPAC-1 and Panc-1, and human umbilical vein endothelial cells (HUVECs) were obtained directly from ATCC (Manassas, VA, USA). These cell lines were periodically authenticated by monitoring cell morphology, growth curve analysis and inspection of mycoplasma contamination, which was examined using a mycoplasma detection kit (Lonza, Rockland, ME, USA). Cells were cultured at 37 °C in a humidified 5% CO_2_ incubator in Dulbecco's modified Eagle's medium containing 10% fetal bovine serum (MiaPaCa-2, Panc-1), Iscove's modified Dulbecco's medium containing 10% fetal bovine serum (CFPAC-1), and M199 medium containing 10% fetal bovine serum, 5 U ml^−1^ heparin and 5 ng ml^−1^ bFGF (HUVECs).

### Preparation of recombinant CTHRC1 and CTHRC1-neutralizing antibody

Recombinant CTHRC1 (rCTHRC1) was prepared as described previously.^7^ A mouse monoclonal antibody against CTHRC1 was generated using rCTHRC1 and contained the IgG2 isotype. The specific binding of the antibody with high affinity to CTHRC1 was determined by enzyme-limked immunosorbent assay (ELISA), and the neutralizing activity was determined by cell-based assays with CTHRC1-producing pancreatic cancer cell lines. A detailed description of the antibody will be published elsewhere.

### *In vivo* animal studies

All animal studies were performed in compliance with the policy of the KRIBB Animal

Care and Use Committee. NOD SCID gamma (NSG) mice (The Jackson Laboratory, Bar Harbor, ME, USA) were used for the implantable tumor studies. For orthotopic xenografts, MiaPaCa-2 or Panc-1 cell lines were suspended at a density of 1 × 10^6^ cells in 100 μl of serum-free culture medium and injected into the body of the pancreas of 5-week-old NSG female mice after exposing the pancreas with an abdominal incision. Five days after implantation, the mice were divided into two groups and treated with the control IgG or the CTHRC1 neutralizing antibody by intraperitonial (i.p.) injection. For Matrigel plug assays, NSG female mice were subcutaneously injected with Matrigel containing control IgG or rCTHRC1. After 7 days, the plugs were removed, photographed, and embedded in optimal cutting temperature (OCT) compound (Sakura, Torrance, CA, USA) for examination of neovessel formation. For TEM migration assays, 1 × 10^6^ Panc-1 cells stably transfected with small hairpin (inhibitory) RNA against CTHRC1 (PANC-1/shCTHRC1) or control shRNA (PANC-1/shCON) were injected into the body of the pancreas of NSG female mice, which was exposed by abdominal incision.

### Proliferation assay

HUVECs in growth media were seeded in gelatin-coated 24-well plates at 2.0 × 10^3^ cells per well. After 24 h in culture, the medium was removed, and the cells were incubated in fresh M199 media for 6 h. Cells were then treated with different concentrations of rCTHRC1 and incubated for 48 h. Cell proliferation was quantified by direct cell counting.

### Migration assay

rCTHRC1-stimulated migration of HUVECs was assayed using a modified Boyden chamber method employing Chemotaxicells (Kurabo, Osaka, Japan) lined with polyvinylpyrrolidone filters (pore size: 8 μm diameter). A cell suspension (0.4 ml) containing 2 × 10^5^ cells was introduced into the Chemotaxicells, which were then placed in the wells of a 24-well plate containing 0.8 ml of medium supplemented with rCTHRC1. After incubation for 4 h at 37 °C, Chemotaxicell filters were quantified by counting the number of stained cells under a light microscope (Olympus). All assays were performed in triplicate, and the results were confirmed in three independent experiments.

### Tube-formation assay

HUVECs in 100 μl of EGM medium supplemented with rCTHRC1 (0, 0.1, 0.2, and 0.5 μg ml^−1^) were inoculated at a density of 1 × 10^5^ per well into 96-well plates. The resulting web-like capillary structure was viewed with a microscope under × 100 magnification and captured with an Olympus DPIX70 digital camera (Olympus, Tokyo, Japan). Tube formation was quantified by determining the number of pixels occupied by the tubes in each image using the KS Lite Image program.

### Luciferase assay

HUVECs were transfected with the firefly luciferase reporter plasmid pCRE-Luc, pSRELuc or pAP1-Luc (Stratagene, La Jolla, CA, USA), together with the transfection control plasmid pRL-TK, which expressed *Renilla* luciferase (Promega, Madison, WI, USA), using a Neon electrophoresis system (Invitrogen, CA, USA). After 24 h of culture, cells were starved by incubating with serum-free M199 medium for 6 h and were then treated with 0.5 μg ml^−1^ of recombinant CTHRC1 for 24 and 48 h. Cells were lysed, and luciferase activity was measured using a Dual-Luciferase Reporter Assay system (Promega, Madison, WI, USA).

### Western blot analysis

Whole-cell lysates were prepared in radioimmunoprecipitation assay buffer, and protein concentrations were determined with a bicinchoninic acid protein assay kit (Pierce, Rockford, IL, USA). Samples containing equal amounts of protein were resolved by SDS-polyacrylamide gel electrophoresis (SDS-PAGE) and transferred to Hybond-enhanced chemiluminescence (ECL) nitrocellulose membranes (Bio-Rad, Hercules, CA, USA). The membranes were probed with the appropriate primary antibodies diluted in Tris-buffered saline/Tween-20 containing 5% bovine serum albumin and then incubated with horseradish peroxidase-conjugated secondary antibodies. Bound antibodies were visualized using enhanced chemiluminescence reagents (AbFrontier, Seoul, Korea).

### Enzyme-linked immunosorbent assay

Ang-2 was measured in HUVECs by a sandwich enzyme-linked immunosorbent assay (ELISA) using a human Ang-2 ELISA kit (R&D Systems, Minneapolis, MN, USA).

### Immunofluorescence confocal microscopy

Samples were embedded in OCT compound and snap-frozen. Sections (5-μm thick) were fixed in 4% paraformaldehyde for 15 min. For immunofluorescence staining, frozen sections were blocked with 1% bovine serum albumin and 5% fetal bovine serum. Sections were immunostained for Tie2 using goat polyclonal anti-Tie2 (Santa Cruz Biotechnology, CA, USA) antibodies and for the EC and hematopoietic cell markers CD31 and CD45 using fluorescein isothiocyanate (FITC)-conjugated anti-CD31 and allophycocyanin (APC)-conjugated anti-CD45 antibodies, respectively (BD Pharmingen, CA, USA). Immunohistochemistry images were taken using an Axioskop 2 Plus direct microscope (Zeiss, Oberkochen, Germany) equipped with a Radiance 2100 three-laser confocal device (Bio-Rad, Segrate, Italy) and a W-PI × 10/0.23 or Plan-Neofluor × 20/0.5 numerical aperture objective lens (Zeiss). Images were captured using the AxioCam HRc system and Axiovision 3.1 version 4.4 software (Zeiss). Fluorescent signals from the individual fluorophores were sequentially acquired from single optical sections and were analyzed using Paint Shop Pro X (Corel, Ottawa, Canada).

## Results

### CTHRC1 enhances tumor vascularity and angiogenesis in a non-autonomous manner

**Figure 1:**
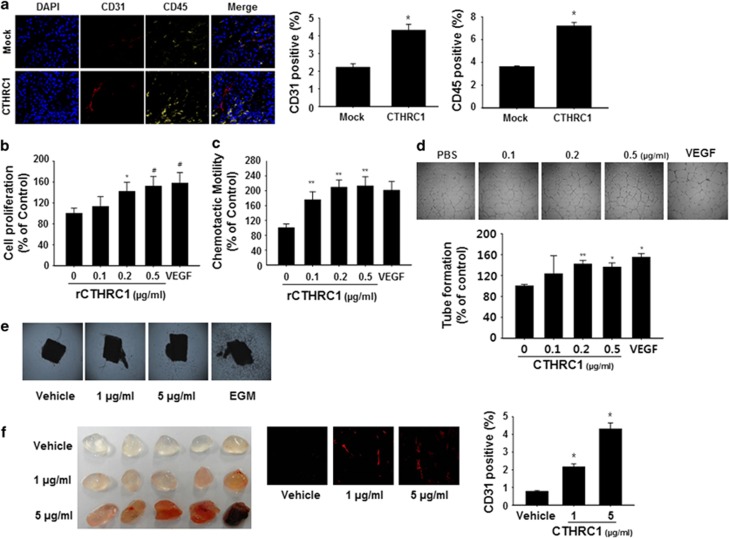
CTHRC1 enhances tumor vascularity and angiogenesis. (**a**) CTHRC1-overexpressing or mock-transfected MiaPaCa-2 cells were orthotopically injected into NSG mice (*n*=6). Four weeks after tumor challenge, mice were killed, and the tumors were excised. The CD31^+^ and CD45^+^ cells in the excised tumors were detected by immunofluorescence analysis using confocal microscopy. Images of one representative mouse per group are shown. Proliferation (**b**), migration (**c**) and capillary-like tube formation (**d**) by HUVECs were assessed after treatment with different concentrations of rCTHRC1 or VEGF (0.02 μg ml^−1^). (**e**) Aortic segments from C57BL/6 mice (five mice per treatment group) were exposed to rCTHRC1 (1 or 5 μg ml^−1^) or a PBS control for 10 days. The experiment was performed twice, and representative images are shown. (**f**) C57BL/6 mice (*n*=5) were injected with 0.6-ml of Matrigel containing vehicle or rCTHRC1 (1 or 5 μg ml^−1^). Seven days after injection, Matrigel plugs were excised from mice. Data are reported as the mean±s.d. (*P<0.01, ^#^*P*<0.05, **P<0.001 versus vehicle). CTHRC1, collagen triple-helix repeat-containing 1; HUVECs, human umbilical vein endothelial cells; PBS, phosphate-buffered saline; rCTHRC1, recombinant CTHRC1; VEGF, vascular endothelial growth factor.

We previously showed that CTHRC1 is an intrinsic factor that promotes tumorigenesis and metastasis in a pancreatic cancer xenograft model.^7^ However, the role of CTHRC1 as an extrinsic factor in the tumor microenvironment and its contribution during tumorigenesis remain unknown. To determine whether CTHRC1 affects tumor-adjacent cells, including immune cells and ECs, during pancreatic cancer development, we orthotopically injected CTHRC1-overexpressing MiaPaCA-2 (MiaPaCa-2/CTHRC1) cells, which had been transfected with a CTHRC1 expression plasmid, into NSG mice. Four weeks after the tumor challenge, we analyzed tumor tissues from MiaPaCa-2/CTHRC1 cell-injected mice for cells positive for CD45, a general lymphohematopoietic cell marker, and CD31, an EC marker, by immunofluorescence. The CD45^+^ and CD31^+^ cell populations were significantly increased in tumor tissues from MiaPaCA-2/CTHRC1 cell-injected mice compared with those from mice injected with MiaPaCa-2/Mock cells (Figure 1a), indicating that CTHRC1 has the potential to modulate vascularity. EC proliferation and chemotaxis are important processes during angiogenesis; thus, we first examined the effects of CTHRC1 on human umbilical vein EC (HUVEC) proliferation and motility *in vitro*. Exposure to rCTHRC1 for 48 h significantly increased the total number of cells and the chemotactic ability in a concentration-dependent manner (Figure 1b and c). Next, we evaluated the effect of CTHRC1 on EC tube formation and EC sprouting in aortic ring segments *in vitro*. Addition of CTHRC1 to the HUVECs induced significant tube formation (Figure 1d) and EC spreading in a concentration-dependent manner (Figure 1e). To further assess the role of CTHRC1 in angiogenesis *in vivo*, we employed Matrigel implant assays in mice. As shown in Figure 1f, rCTHRC1-containing Matrigel plugs showed an intense red color and increased CD31^+^ cells, which were dependent on the concentration of rCTHRC1, indicating that CTHRC1 activated ECs, causing microvessel outgrowth and blood vessel formation. Taken together, these results indicate that CTHRC1 has potent angiogenic activity that leads to the activation of ECs.

### CTHRC1 acts through the ERK signaling cascade to induce Ang-2 expression in ECs

**Figure 2:**
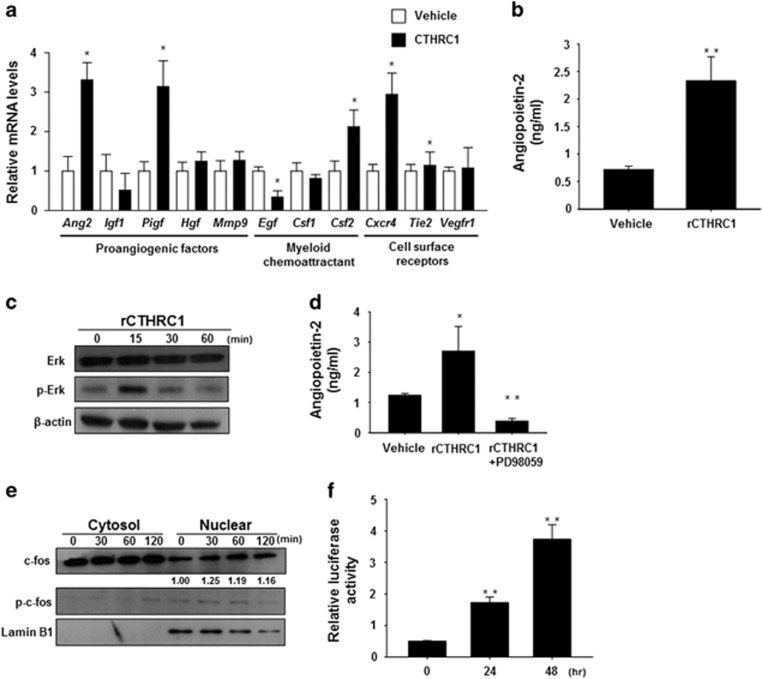
CTHRC1 acts through the ERK signaling cascade to induce Ang-2 expression in ECs. (**a**) Gene expression in rCTHRC1-treated HUVECs was analyzed by RT-qPCR. Differentially expressed genes between the rCTHRC1 and vehicle groups are reported as the mean±s.d. (*P<0.01 versus vehicle). (**b**) HUVECs were treated with rCTHRC1 (0.5 μg ml^−1^), and Ang-2 secreted into the culture supernatant was measured by ELISA. Data are reported as the mean±s.d. (**P<0.001 versus vehicle). (**c**) HUVECs were stimulated with rCTHRC1 (0.5 μg ml^−1^) for the indicated times. Whole-cell lysates were prepared and used for Western blotting with antibodies against ERK, p-ERK and actin. Representative images from three independent experiments are shown. (**d**) HUVECs were co-stimulated with the ERK inhibitor PD98059 (100 μM) and rCTHRC1 (0.5 μg ml^−1^) for 18 h, and secreted Ang-2 in the culture supernatants was measured by ELISA. (**e**) Nuclear fractions were collected from HUVECs treated with rCTHRC1 for the indicated times, after which c-fos, p-c-fos and lamin (positive control for the nuclear fraction) were detected by western blotting. Representative images from three independent experiments are shown. (**f**) HUVECs were transfected with AP-1-luc and pRLnull and treated with rCTHRC1 for the indicated times. Dual luciferase activities were determined after treatment, as described in the Materials and Methods section. The data represent firefly luciferase activity normalized to *Renilla* luciferase activity present in each sample. Data are reported as the mean±s.d. (*P<0.01, ^#^*P*<0.05, **P<0.001 versus vehicle). CTHRC1, collagen triple-helix repeat-containing 1; EC, endothelial cell; ELISA, enzyme-linked immunosorbent assay; rCTHRC1, recombinant CTHRC1; RT-qPCR, reverse transcription quantitative PCR.

To elucidate the molecular mechanisms responsible for the activation of ECs by CTHRC1, we investigated the angiogenic factors that are regulated by CTHRC1. To this end, we analyzed the expression of angiogenesis-associated and myeloid cell growth factor/chemoattractant genes by quantitative PCR in HUVECs activated by CTHRC1 (Figure 2a). These analyses showed that CTHRC1 upregulated the messenger RNA (mRNA) levels of the proangiogenic genes *ang-2*, *hgf* (hepatocyte growth factor) and *mmp9* (matrix metallopeptidase 9) in HUVECs. Because CTHRC1 enhanced infiltration of hematopoietic lineage cells into the tumor tissues

(Figure 1a), we focused on myeloid chemoattractant molecules in rCTHRC1-activated ECs. rCTHRC1 upregulated Ang-2 in HUVECs, whereas it had no effect on other myeloid chemoattractant or cell surface receptors, indicating that Ang-2 is a CTHRC1-regulated gene in ECs. Consistent with these results, an analysis of the supernatants from rCTHRC1-treated HUVECs by ELISA confirmed that Ang-2 protein levels were also increased by rCTHRC1 (Figure 2b). Because Ang-2 expression is induced through AP-1 and Ets1 transcriptional factors in response to ERK pathway signaling,^15^ we evaluated whether CTHRC1 induced ERK-mediated activation of AP-1 in ECs. As expected, rCTHRC1 increased the level of phosphorylated ERK in HUVECs (Figure 2c). Treatment with the ERK inhibitor PD98059 reduced the expression of Ang-2 in rCTHRC1-treated HUVECs (Figure 2d), indicating that CTHRC1 regulates Ang-2 expression via the ERK signaling cascade. Next, to identify the AP-1 component responsible for the ERK-dependent response to CTHRC1 in ECs, we assessed phosphorylated c-fos in the nuclear fraction of CTHRC1-treated cells by Western blot analysis. Treatment of HUVECs with rCTHRC1 resulted in the accumulation of phosphorylated c-fos in the nucleus within 30 min (Figure 2e), suggesting that CTHRC1 regulates Ang-2 expression via ERK-dependent AP-1 activation in ECs. To further confirm these findings, we examined the transcriptional activity of AP-1 using a luciferase reporter construct containing an AP-1 binding element following treatment with CTHRC1. As shown in Figure 2f, rCTHRC1 treatment induced a time-dependent increase in luciferase activity in transfected HUVECs. Taken together, these data indicate that CTHRC1 regulates Ang-2 expression through ERK-dependent AP-1 activation in ECs.

### CTRHC1 enhances infiltration of TEMs into pancreatic cancer tissues

**Figure 3:**
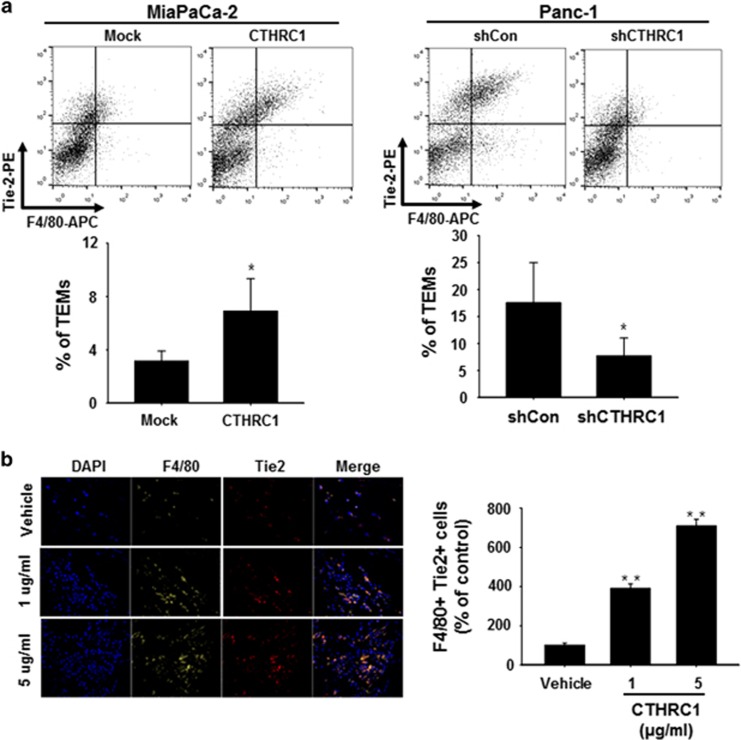
CTRHC1 enhances infiltration of TEMs into pancreatic cancer tissues. (**a**) CTHRC1-overexpressing or mock-transfected MiaPaCa-2 cells (left panel) and 10^5^ shCon- or shCTHRC1-expressing Panc-1 cells (right panel) were orthotopically injected into nude mice (*n*=5). Four weeks after tumor challenge, the percentage of infiltrated TEMs was determined by flow cytometry analysis of cells immunostained for Tie2 and F4/80. Data are reported as the mean±s.d. (*P<0.01, ^#^*P*<0.05, **P<0.001 versus shCon or mock-transfected cells). (**b**) Matrigel containing PBS (control) or rCTHRC1 (1 or 5 μg ml^−1^) was injected into C57BL/6 mice (*n*=5). Seven days after injection, Matrigel plugs were excised and stained for infiltrating TEMs using anti-F4/80 and anti-Tie2 antibodies. TEMs were quantified by counting the number of F4/80 and Tie2 double-positive cells per field for each Matrigel plug. Data are reported as the mean±s.d. (**P<0.001 versus vehicle). CTHRC1, collagen triple-helix repeat-containing 1; PBS, phosphate-buffered saline; TEMs, Tie2-expressing monocytes.

Ang-2 binds the endothelial-specific receptor tyrosine kinase Tie2 and promotes infiltration of Tie2-expressing circulating monocytes into tumor tissues, a step required for the formation of tumor blood vessels.^16^ On the basis of this report, we hypothesized that upregulation of Ang-2 by CTHRC1 in ECs promotes infiltration of TEMs into pancreatic cancer tissues. To test this hypothesis, we first orthotopically injected MiaPaCa-2/CTHRC1 or PANC-1/shCTHRC1 cells into NSG mice and then analyzed the population of TEMs in tumor tissues by flow cytometry. As shown in Figure 3a, the population of TEMs (F4/80 and Tie2 double-positive cells) was increased in tumor tissues from mice injected with MiaPaCa-2/CTHRC1 cells compared with those from mice injected with MiaPaCa-2/Mock cells. For loss-of-function studies, the Panc-1 cell line, which expresses high levels of CTHRC1, was stably transfected with a CTHRC1-specific shRNA, generating the cell line Panc-1/shCTHRC1. Tumors from Panc-1/shCTHRC1-injected mice showed significantly decreased infiltration of TEMs compared with those from mice injected with Panc-1/shCON cells, indicating that CTHRC1-induced Ang-2 enhances infiltration of TEMs into tumor tissues. To further confirm that CTHRC1 enhances infiltration of TEMs, we implanted mice with Matrigel plugs containing different concentrations of rCTHRC1 and then analyzed TEM infiltration into the plugs by confocal microscopy. As shown in Figure 3b, the infiltration of TEMs in rCTHRC1-containing Matrigel plugs was significantly increased compared with that in vehicle-containing plugs, an effect that was concentration dependent. These results suggest that CTHRC1 enhances infiltration of TEMs to the tumor site.

### A CTHRC1-neutralizing antibody inhibits rCTHRC1-induced activation of ECs and infiltration of TEMs in pancreatic cancer xenograft models

**Figure 4:**
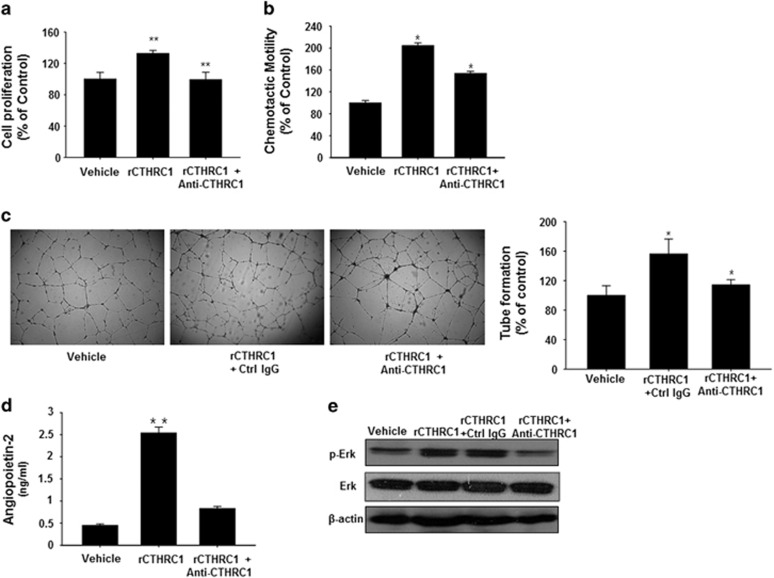
A CTHRC1-neutralizing antibody reduces angiogenesis *in vitro* by abrogating EC activation. Proliferation (**a**), migration (**b**) and capillary-like tube formation (**c**) by HUVECs were assessed after co-treatment with a CTHRC1-neutralizing antibody (10 μg ml^−1^) and rCTHRC1 (0.5 μg ml^−1^). (**d**) HUVECs were co-treated with a CTHRC1-neutralizing antibody (10 μg ml^−1^) and rCTHRC1 (0.5 μg ml^−1^) for 16 h. Secreted Ang-2 in the culture supernatants was measured by ELISA. Data are reported as the means±s.d. (**e**) HUVECs were stimulated with a CTHRC1-neutralizing antibody (10 μg ml^−1^) and rCTHRC1 (0.5 μg ml^−1^). Whole-cell lysates were prepared and used for western blotting with antibodies against ERK, p-ERK and actin. Representative images from three independent experiments are shown. CTHRC1, collagen triple-helix repeat-containing 1; EC, endothelial cell; ELISA, enzyme-limked immunosorbent assay; HUVECs, human umbilical vein endothelial cells.

To further examine the regulatory function of CTHRC1 in EC activation, we used a CTHRC1-neutralizing monoclonal antibody. Treatment with the CTHRC1-specific antibody suppressed CTHRC1-induced EC activation, as shown by the decrease in the total number of HUVECs (Figure 4a) and the diminished chemotactic motility and capillary-like tube formation (Figure 4b and c) after 48 h compared with the control IgG-treated cells. These results suggest that EC angiogenesis and vascular permeability could be effectively suppressed by targeting CTHRC1. Next, using ELISAs, we investigated the level of secreted Ang-2 in the culture supernatants of rCTHRC1-treated HUVECs incubated with or without the CTHRC1-neutralizing antibody. The level of Ang-2 protein was significantly decreased in the culture medium from antibody-treated HUVECs (Figure 4d). As expected, given that CTHRC1-induced Ang-2 expression is responsible for ERK activation by CTHRC1, the levels of phosphorylated ERK were also decreased in rCTHRC1-treated HUVECs incubated with the CTHRC1-neutralizing antibody (Figure 4e).

**Figure 5:**
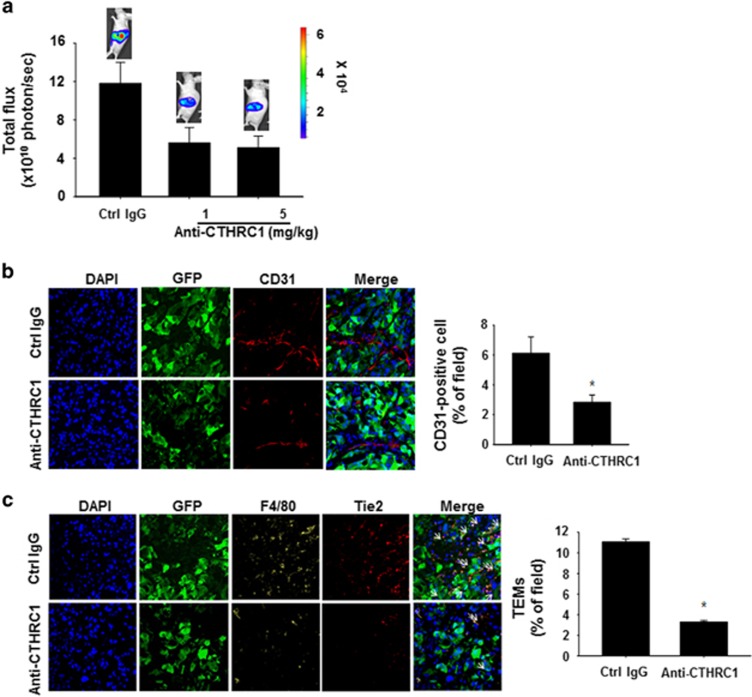
A CTHRC1-neutralizing antibody reduces tumor growth and infiltration of TEMs in pancreatic cancer. (**a**) CFPAC-1 cells stably expressing a firefly luciferase gene and a GFP gene were orthotopically injected into C57BL/6 mice (*n*=5). Five days after injection of the tumor cells, tumor-bearing mice were administered control IgG (5 mg kg^−1^) or the CTHRC1-neutralizing antibody (5 mg kg^−1^) intraperitoneally twice a week. *In vivo* tumor growth was monitored by measuring the total flux from bioluminescence imaging. Four weeks after the tumor challenge, the total flux on the left side of the body was measured. (**b**) Primary tumors derived from CFPAC-1 cells were used for immunofluorescence staining of CD31^+^ cells. CD31^+^ cells in the excised tumors were detected by immunofluorescence analysis using confocal microscopy. The bar graph shows the quantification of CD31^+^ cells in the experimental group tumors compared with that of the control group tumors. Data are reported as the mean±s.d. (*P<0.01 versus rCTHRC). (**c**) Primary tumors of CFPAC-1 were excised and stained for infiltrating TEMs using anti-F4/80 and anti-Tie2 antibodies. The bar graph shows the quantification of infiltrated TEMs in the experimental group tumors compared with that of the control group tumors. Data are reported as the mean±s.d. (*P<0.01 versus rCTHRC). CTHRC1, collagen triple-helix repeat-containing 1; Ig, immunoglobulin; rCTHRC, recombinant CTHRC.

Finally, using luminescence-based orthotopic pancreatic tumor xenograft models, we examined the therapeutic potential of the CTHRC1-neutralizing antibody. CFPAC-1-luc/GFP cells, which expressed the firefly luciferase gene and the GFP gene, were implanted into the pancreas of C57BL/6 mice, and the antibody (1 and 5 mg kg^−1^) was administered twice a week beginning 7 days after the injection of tumor cells. At 4 weeks post-implantation, antibody-treated mice showed reduced tumor growth compared with the control IgG-treated mice (Figure 5a). In addition, an immunofluorescence analysis demonstrated substantially reduced blood vessel density in xenograft tumor tissues from mice injected with the CTHRC1-neutralizing antibody (Figure 5b). Infiltration of TEMs into the tumor tissues was also reduced in mice administered antibodies compared with those administered control IgG (Figure 5c). Thus, in our experimental mouse model, a CTHRC1-neutralizing antibody exhibited therapeutic potential for treatment of pancreatic cancer through inhibition of vascular processes.

## Discussion

Here, we demonstrated that in the pancreatic tumor microenvironment, the CTHRC1/Ang-2/TEM axis can be a target for anti-tumor therapy by reducing tumor angiogenesis. CTHRC1 was initially identified in a screen for differentially expressed genes in balloon-injured versus normal rat vessels.^17^ Recent studies have demonstrated that aberrant expression of CTHRC1 is associated with tumor malignance in a variety of cancers, including breast cancer and gastric cancer.^18, 19, 20, 21^ We also reported that CTHRC1 expression is correlated with the progression and metastasis of pancreatic cancer.^7^

The expression of CTHRC1 is known to be regulated by several factors. A group of microRNAs that inhibit CTHRC1 were reported to block tumor growth.^22, 23^ Currently, factors such as demethylation of the CTHRC1 promoter and canonical Wnt signaling are known to upregulate CTHRC1.^24, 25, 26^ However, the regulation and function of CTHRC1 in the tumor microenvironment is largely unknown. A further important step is to identify the mechanism or mechanisms of how CTHRC1 is regulated and its role in the tumor microenvironment.

Because CTHRC1 is known to have a role in vascular remodeling through matrix deposition and cell migration in injured tissues,^27, 28, 29^ we proposed that CTHRC1 may be a novel paracrine factor in the regulation of neovasculogenesis in the tumor microenvironment. Our results provide strong experimental evidence that CTHRC1 promotes angiogenesis and vascular permeability in a paracrine manner. Immunofluorescence analyses showed that CTHRC1 overexpression is correlated with microvessel density in mouse tumor models. Tumors derived from CTHRC1-overexpressing cells exhibited significantly enhanced vascular density compared with those formed by control (mock-transfected), and exogenous application of rCTHRC1 promoted vessel sprouting from explanted rat aortic rings and neovascularization in Matrigel plugs. We also demonstrated that rCTHRC1 increased EC proliferation, migration, and tubular network formation (Figure 1). Moreover, rCTHRC1 induced expression of several factors involved in neovasculogenesis in ECs. We focused on Ang-2 expression among CTHRC1-induced proangiogenic factors because CD45^+^ cell infiltration into the tumor tissues is increased and because CD45^+^ cells of the monocytic lineage comprise the largest and most heterogeneous group of bone marrow-derived cells that function as vascular modulators.^30^ The majority of CD45^+^ cells was F4/80^+^ TAMs and TEMs, which express Tie2 and thus are able to respond to Ang-2. Because Ang-2 is a chemoattractant for human and murine TEMs *in vitro* and *in vivo*,^14^ we hypothesized that rCTHRC1-induced Ang-2 expression enhanced infiltration of CD45^+^ cells into tumor tissues. We next determined how CTHRC1 regulates Ang-2 in ECs. CTHRC1 regulates MMP9 expression in colon cancer through ERK activation.^31^ Indeed, we found that Ang-2 expression was regulated by ERK-dependent activation of AP-1 and Ets-1, which have important roles in early blood vessel remodeling and promotion of angiogenesis.^15^ Consequently, we hypothesized that Ang-2 expression is regulated by CTHRC1-induced ERK/AP1 signaling in ECs. To test this hypothesis, we investigated ERK/AP-1 signaling-mediated regulation of Ang-2 by CTHRC1. As predicted, rCTHRC1 induced activation of the ERK signaling pathway followed by nuclear translocation of AP-1 and a subsequent increase in Ang-2 mRNA levels (Figure 2). Blockade of ERK activation with specific inhibitors prevented CTHRC1-induced Ang-2 secretion. These results suggest that ERK pathways promote angiogenesis by inducing the expression of Ang-2, at least in part through AP-1 (Figure 2). In addition, the Ras/Raf/MEK/ERK pathway is required for EC function during angiogenesis. For example, ERK activation in primary ECs results in increased cell proliferation and migration, and paxillin and focal adhesion kinase are expressed at higher levels in ECs in which the ERK signaling cascade is activated, enhancing cytoskeletal organization and increasing cell motility.^32, 33^ Collectively, our data support the novel finding that CTHRC1-enhanced ERK activation regulates not only Ang-2 expression but also proliferation and migration of ECs.

Myeloid cells, which are essential for blood vessel formation and function in tumors,^34^ are considered paracrine factors that are regulated by CTHRC1. Because myeloid cells, such as monocytes/macrophages, can trigger vessel growth by releasing angiogenic factors, such as Ang2,^35^ TEMs, among the most proangiogenic subsets of myeloid cells,^12^ which express Tie2, could be a target of CTHRC1. TEMs are known to be involved in tumor vascularization^14, 36, 37^ and are attracted by Ang-2 in tumors, suggesting that CTHRC1-induced upregulation and release of Ang-2 serve to recruit TEMs into the tumor tissues. Consistent with this interpretation, our data showed that CTHRC1 expression associated with tumor angiogenesis resulted in greater infiltration of murine TEMs into the tumors (Figure 3), which suggests that Ang-2 regulates TEMs by the increase in blood vessels in CTHRC1-overexpressing tumors.

A CTHRC1-neutralizing antibody, developed as a tool for functional studies, markedly reduced tumor growth and tumor vasculature in CTHRC1-expressing tumors (Figure 5). In addition, the antibody also reduced the expression and release of Ang-2 in the ECs and inhibited infiltration of TEMs in CTHRC1-overexpressing tumor tissues, consistent with the inhibition of the proangiogenic programs of TEMs in murine tumors by Ang-2 blockade.^38^ Collectively, our findings suggest that the CTHRC1-neutralizing antibody has the potential to treat not only pancreatic cancer but also CTHRC1-overexpressing cancers, such as colon cancer, breast cancer and melanoma.

**Figure 6:**
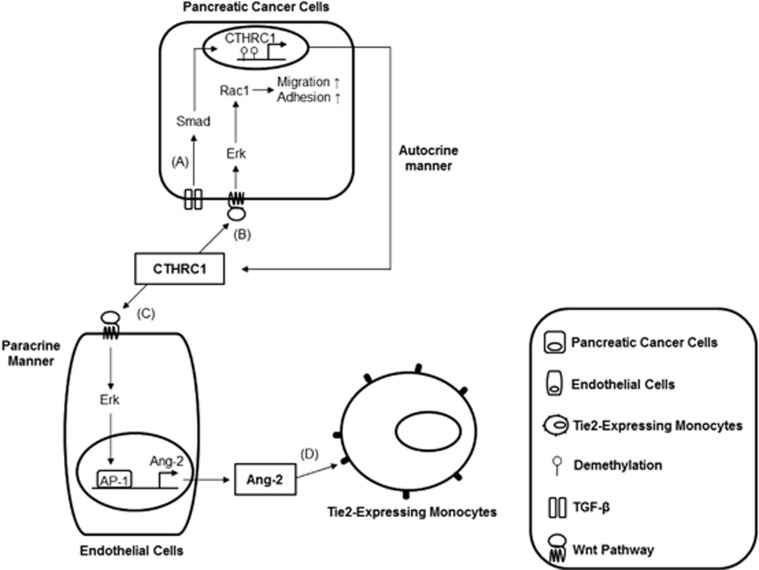
Schematic diagram of CTHRC1 function in the tumor microenvironment. (A) CTHRC1 is upregulated by promoter demethylation and TGF-β-mediated Smad signaling. (B) CTHRC1 acts in an autocrine manner to promote tumor cell migration and adhesion via the non-canonical Wnt-mediated Rac1 pathway. (C) CTHRC1 acting in a paracrine manner activates ECs through the non-canonical Wnt pathway in human ECs. Activation of the noncanonical Wnt pathway regulates Ang-2 via the ERK-AP1 signaling cascade. (D) TEMs migrate to Ang-2–expressing tumor regions. CTHRC1, collagen triple-helix repeat-containing 1; TEMs, Tie2-expressing monocytes; TGF, transforming growth factor.

Previously, CTHRC1 has induced the migratory and adhesiveness of pancreatic cancer cells through Wnt-Rac1 pathway in an autocrine manner.^7^ Here we provide compelling evidence for a regulatory effect of angiogenesis by CTHRC1 in paracrine manner. Ang-2 secreted by CTHRC1-activated ECs could directly promote angiogenesis. CTHRC1-activated ECs could also regulate angiogenesis indirectly by promoting infiltration of TEMs in response to secreted Ang-2. These findings demonstrate that increased CTHRC1 levels in the tumor microenvironment may directly lead to EC activation and infiltration of the TEMs; thus, implying CTHRC1 as an important contributor to tumor angiogenesis. Collectively, we described schematic illustration in regard to the function of CTHRC1 within tumor microenvironment in Figure 6.

Finally, our results suggest that CTHRC1 may be a promising therapeutic target for treatment of angiogenesis-dependent vascular diseases.
